# Is too much work engagement detrimental? Linear or curvilinear effects on mental health and job performance

**DOI:** 10.1371/journal.pone.0208684

**Published:** 2018-12-26

**Authors:** Akihito Shimazu, Wilmar B. Schaufeli, Kazumi Kubota, Kazuhiro Watanabe, Norito Kawakami

**Affiliations:** 1 Center for Human and Social Sciences, College of Liberal Arts and Sciences, Kitasato University, Kanagawa, Japan; 2 Asia Pacific Centre for Work Health and Safety, University of South Australia, Adelaide, Australia; 3 Research Unit Occupational & Organizational Psychology and Professional Learning, KU Leuven, Leuven, Belgium; 4 Department of Social and Organizational Psychology, Utrecht University, Utrecht, The Netherlands; 5 Department of Biostatistics, Yokohama City University School of Medicine, Kanagawa, Japan; 6 Department of Mental Health, The University of Tokyo Graduate School of Medicine, Tokyo, Japan; University of East Anglia, UNITED KINGDOM

## Abstract

Most studies report a positive relationship of work engagement with health and job performance, but, occasionally, a “dark side of engagement” has also been uncovered. The current study examined two hypotheses: whether work engagement has (1) a U-shaped curvilinear relation with psychological distress and (2) an inverted U-shaped curvilinear relation with job performance (i.e., in-role performance and creative behavior). A two-wave longitudinal Internet survey with a time lag of seven months was conducted among 1,967 Japanese employees. To test our hypotheses, we used a two-wave panel design and examined the lagged and concurrent relations between work engagement and both outcomes. The results confirmed that work engagement had a curvilinear relation with psychological distress concurrently; a favorable effect was found initially, but this disappeared at intermediate levels of work engagement, and, at higher levels, an adverse effect became prominent. In addition, work engagement had a curvilinear relation with in-role performance both concurrently and longitudinally; the higher the levels of work engagement, the stronger the favorable effects on in-role performance. However, contrary to our expectations, work engagement had a linear relation with psychological distress longitudinally and with creative behavior both concurrently and longitudinally. Hence, our results suggest that work engagement plays a different role in health enhancement compared to performance enhancement. Leveling-off and adverse effects of high work engagement were observed for psychological distress in the short and not in a long run. In contrast, no leveling-off effect of high work engagement was observed for job performance. Thus, except for the short-term effect on psychological distress, no dark side of work engagement was observed for psychological distress and job performance.

## Introduction

A host of studies suggests that work engagement, which is defined as “a positive, fulfilling, work-related state of mind that is characterized by vigor, dedication, and absorption” [1], is beneficial in nature. For instance, work engagement has been associated with better mental and physical health in terms of low levels of depression [2,3] and anxiety [4], healthy cardiac autonomic activity [5], better cortisol suppression in response to dexamethasone [6], better sleep quality [7,8], and less psychological distress [9]. In addition, research also suggests that work engagement is beneficial for employees’ job performance, and, hence, for organizations. This is illustrated by the fact that work engagement is related to low sickness absence frequency [10], low risk of long-term sickness absence [11], self-rated job performance [12], manager’s and co-workers’ rated job performance [13], task- and contextual performance [14], innovativeness [15,16], creativity [17], high financial returns [18], good service quality [19], fewer errors [20], superior business outcomes such as high productivity and profitability [21], and business growth [16].

So far, virtually all studies on the relationships between work engagement and indicators of health and job performance assume that these relations are linear in nature (the only exception was Caesens et al. [22], who examined the curvilinear relations). This means that when levels of engagement are increasing (or decreasing) levels of health and job performance are increasing (or decreasing) at the same rate. However, some researchers have suggested that there might be a dark side of engagement as well [23, 24, 25, 26]. According to Sonnentag [26], work engagement might show a curvilinear relationship with positive outcomes. Specifically, up to a certain level, work engagement might be positively related to these outcomes, but, at high levels, it might become detrimental [26]. So the question arises, is there an upper limit of favorable effect of work engagement, or can “over-engagement” have negative consequences for health and job performance? Based on the previous overview, the current paper challenges the assumption of linearity and investigates curvilinear relationships between work engagement on the one hand and health and job performance on the other using two-wave longitudinal data. This study focused on psychological distress as an indicator of health and in-role performance and creative behavior as indicators of job performance, because previous studies that investigated linear effect of work engagement in cross-sectional and longitudinal designs focused on those three outcomes intensively [27, 28, 29].

Two approaches may be used to build our argument of curvilinearity: the meta-theoretical principle of too-much-of-a-good-thing effect (TMGT) [30] and Warr’s [31] Vitamin Model. The TMGT effect “occurs when ordinarily beneficial antecedents reach inflection points after which their relations with desired outcomes cease to be linear and positive, instead yielding an overall curvilinear pattern” ([30], p. 316). In line with this effect, Caesens et al. [22] examined the curvilinear relationship between work engagement and turnover intentions. Based on cross-sectional data across two different samples, they found that an additional increase of work engagement did not provide additional desirable effect beyond a certain level [22].

According to Warr [31], mental health is affected by environmental psychological features such as job characteristics in a way that is analogous to the non-linear effects that vitamins are supposed to have on our physical health. Warr [31] argues that continued intake of vitamins may lead to two different kinds of effects: (1) a Constant Effect (CE), whereby health does not improve and no noxious consequences are observed, and (2) an Additional Decrement (AD), whereby an overdose of vitamins leads to a toxic concentration in the body (i.e., poor bodily functioning and ill-health). Warr [31, 32] assumes that six job characteristics (i.e., job autonomy, job demands, social support, skill utilization, skill variety, and task feedback) have effects similar to vitamins A and D (i.e., AD effect). The remaining three job characteristics (i.e., salary, safety, and task significance) are supposed to follow the CE pattern (CE effect). Although the model is basically connected with health outcomes, the nine job characteristics in the model can be classified into job demands or job resources which connect with job performance through work engagement [33]. Therefore, we will adopt the model also to job performance.

There seem to be two possible pathways through which over-engagement may have detrimental effects on health and job performance. The first is a *behavioral* pathway whereby excessive amounts of time and effort are spent at work [34] and, consequently, job demands and work–home interference are increasing and opportunities to recover from work are insufficient [26]. The second is a *psychophysiological* pathway whereby a continuously high level of arousal leads to sustained activation [35] with negative side-effects such as psychological distress. This is illustrated in a recent study on work engagement and C-reactive protein [36], which is a risk factor for cardiovascular disease. This study shows that lower *and* higher levels of engagement are related to higher levels of C-reactive protein. These two pathways are in line with Cohen’s costs of coping theory [37] and Hockey’s cognitive-energetical framework [38] that explain impaired performance as well as health”.

Taken together, these indicate that work engagement shows a curvilinear relationship with outcomes like health and job performance. This means that, up to a certain level, work engagement might have favorable effects on these outcomes, but, at higher levels, it might become detrimental and lead to deteriorated health and impeded job performance [39].

More specifically, we formulate three hypotheses:

1. Work engagement has a lagged and curvilinear relationship with psychological distress following a U-shaped pattern; both lower *and* higher levels of work engagement are associated with higher future psychological distress (Hypothesis 1a).
2. Work engagement has a lagged and curvilinear relationship with in-role performance following an inverted U-shaped pattern; both lower *and* higher levels of work engagement are associated with poorer future in-role performance (Hypothesis 2a).
3. Work engagement has a lagged and curvilinear relationship with creative behavior following an inverted U-shaped pattern; both lower *and* higher levels of work engagement are associated with poorer future creative behavior (Hypothesis 3a).

According to van Dierendonck et al. [40], if the “true” effect of time is much shorter than the time lag of the study, a model with a concurrent effect will represent the longitudinal data more adequately than a model with a lagged effect in the same direction (for more information, see Zapf et al. [41]). Therefore, we also formulated the following hypotheses to examine the concurrent relation between work engagement and outcomes. More specifically:

4 Work engagement has a concurrent and curvilinear relationship with psychological distress following a U-shaped pattern (Hypothesis 1b).
5 Work engagement has a concurrent and curvilinear relationship with in-role performance following an inverted U-shaped pattern (Hypothesis 2b).
6 Work engagement has a concurrent and curvilinear relationship with creative behavior following an inverted U-shaped pattern (Hypothesis 3b).

## Materials and methods

### Procedure and participants

This study was a part of a larger research project on socioeconomic status and health. A prospective survey was conducted among research volunteers who were registered at an Internet survey company in Japan. The Internet survey system did not allow missing values, and, therefore, respondents had to respond to all questions. The current study used the data obtained in two waves. For the first-wave survey, a total of 13,564 employees volunteered who corresponded in age, gender, and resident area to a Japanese representative sample. They were randomly invited to participate. The recruitment stopped after the number of participants reached 2,520 as the research budget was exhausted. After seven months, all respondents who completed the first-wave survey (N = 2,520) were invited to the second wave. A total of 553 respondents dropped out (21.9%), resulting in 1,967 respondents being included in the current study. The survey company matched respondents’ survey responses over time according to the participants’ IDs. Informed consent was obtained from all participants included in the study.

The mean age of the participants was 45.3 (SD = 12.5). Of the participants, 51.2% were male, 63.7% were married, and 44.5% had a university degree. Over half of the participants had a full-time, tenured labor contracts (53.6%). Most participants were white collar workers (84.7%) : 26.6% of participants were employed in clerical jobs, 25.3% in technical and engineering jobs, 13.4% in service jobs, 11.5% in sales and marketing jobs, and 8.0% in managing jobs. Compared to the Japanese population, our sample was more highly educated [42].

To examine potential selection bias, we compared “completers,” who answered both first- and second-wave surveys (N = 1,967), with “dropouts,” who answered only the first-wave survey (N = 553), with respect to their baseline demographic characteristics and their scores on the study variables at Time 1 (T1). The completers were significantly older (Mean 45.3, SD = 12.5 vs. Mean 41.5, SD = 13.7; Welch’s *t* [826.64] = 5.79, *p* < 0.001) than dropouts. There were also differences between the two groups regarding gender (χ^2^ [1] = 6.19, *p* < 0.05). Specifically, more men completed the survey (51.2%) as compared to the dropouts (45.2%). Thus, relative to dropouts, our sample is older and includes more men. The procedures of the investigation were approved by the ethics review board of The University of Tokyo before starting the study (No.3158).

### Measures

#### Work engagement

Work engagement was assessed using the short form of the Utrecht Work Engagement Scale (UWES) [1, 43]. The UWES includes three subscales that reflect the underlying dimensions of engagement: Vigor (3 items; e.g., “At my job, I feel strong and vigorous”), Dedication (3 items; e.g., “I am enthusiastic about my job”), and Absorption (3 items; e.g., “I am immersed in my work”). Each item was scored on a seven-point Likert scale ranging from 0 (“never”) to 6 (“always”). According to the validation study of the Japanese version of the UWES [43], it is recommended that work engagement be treated as a unitary construct due to the high correlations among the three components. Therefore, the sum of the scores of all nine items was used. Cronbach’s alpha coefficients were 0.95 for T1 and 0.96 for Time 2 (T2). The correlation between T1 and T2 engagement scores was 0.76 (*p* < 0.001).

#### Psychological distress

Psychological distress was assessed using the corresponding subscales of the Brief Job Stress Questionnaire (BJSQ) [44]. Psychological distress was assessed by means of 15 items, mainly reflecting irritability, fatigue, anxiety, and depression. Each item was scored on a four-point Likert scale ranging from 1 (“almost never”) to 4 (“almost always”). Cronbach’s alpha coefficients were 0.95 for T1 and 0.95 for T2. The correlation between T1 and T2 distress score was 0.66 (*p* < 0.001).

#### Job performance

In-role performance, which refers to work behaviors that directly serve the goals of the organization, was assessed by two items from Williams and Anderson’s scale [45] (e.g., “I adequately complete assigned duties”). Each item was scored on a four-point Likert scale ranging from 1 (“disagree”) to 4 (“agree”). The correlations between the items were .71 (*p* < 0.001) for T1 and .72 (*p* < 0.001) for T2, respectively. The correlation between T1 and T2 performance scores was 0.46 (*p* < 0.001). Creative behavior, which refers to the production of novel and useful ideas, was assessed by three items from George and Zhou’s scale [46] (e.g., “I am a good source of creative ideas”). Each item was scored on a four-point Likert scale ranging from 1 (“disagree”) to 4 (“agree”). Cronbach’s alpha coefficients were 0.91 for T1 and 0.91 for T2. The correlation between T1 and T2 creativity scores was 0.61 (*p* < 0.001).

### Statistical analyses

First, we conducted confirmatory factor analyses to examine the discriminant validity of the constructs of interest in each survey (T1 and T2). Specifically, we compared a four-factor model, which assumes that each construct (i.e., work engagement, psychological distress, in-role performance, and creative behavior) is independent, although correlated, to the one-factor model, which assumes that all subscales measuring the four constructs load on one general factor. Following the recommendation of Coffman & MacCallum [47], we used parcels (i.e., subscales) instead of individual items as indicators of the latent variables. According to Coffman & MacCallum [47], parceling may have some disadvantages in terms of misspecifying models, obscuring additional unmodeled factors, and decrease in convergence to proper solutions. Nevertheless, they claim, that parceling has several advantages as well such as higher reliability of the indicators, reduced number of measured variables in a model, and providing an alternative to data transformations or alternative estimation techniques when working with nonnormally distributed variables.

Next, to test our hypotheses, we used a so-called two-wave panel model [41], as described by van Dierendonck et al. [40]. This analysis combines “exploring longitudinal directions using panel analysis with structural equation models” [48] and “investigating curvilinear relationships using multiple regression” [49]. According to van Dierendonck et al. [40], the advantages of this approach are twofold. First, it provides a statistical test that allows for directional conclusions. Second, structural equation models can also reveal concurrent relations between variables.

Using AMOS software package [50], we tested six structural equation models—three lagged and three concurrent models for each outcome (i.e., psychological distress, in-role performance, and creative behavior). By comparing the fit of each of these models with that of the stability model, we can determine the most likely direction, shape (linear or curvilinear), and time frame (concurrent or seven months lagged) of the effect.

**Fig 1:**
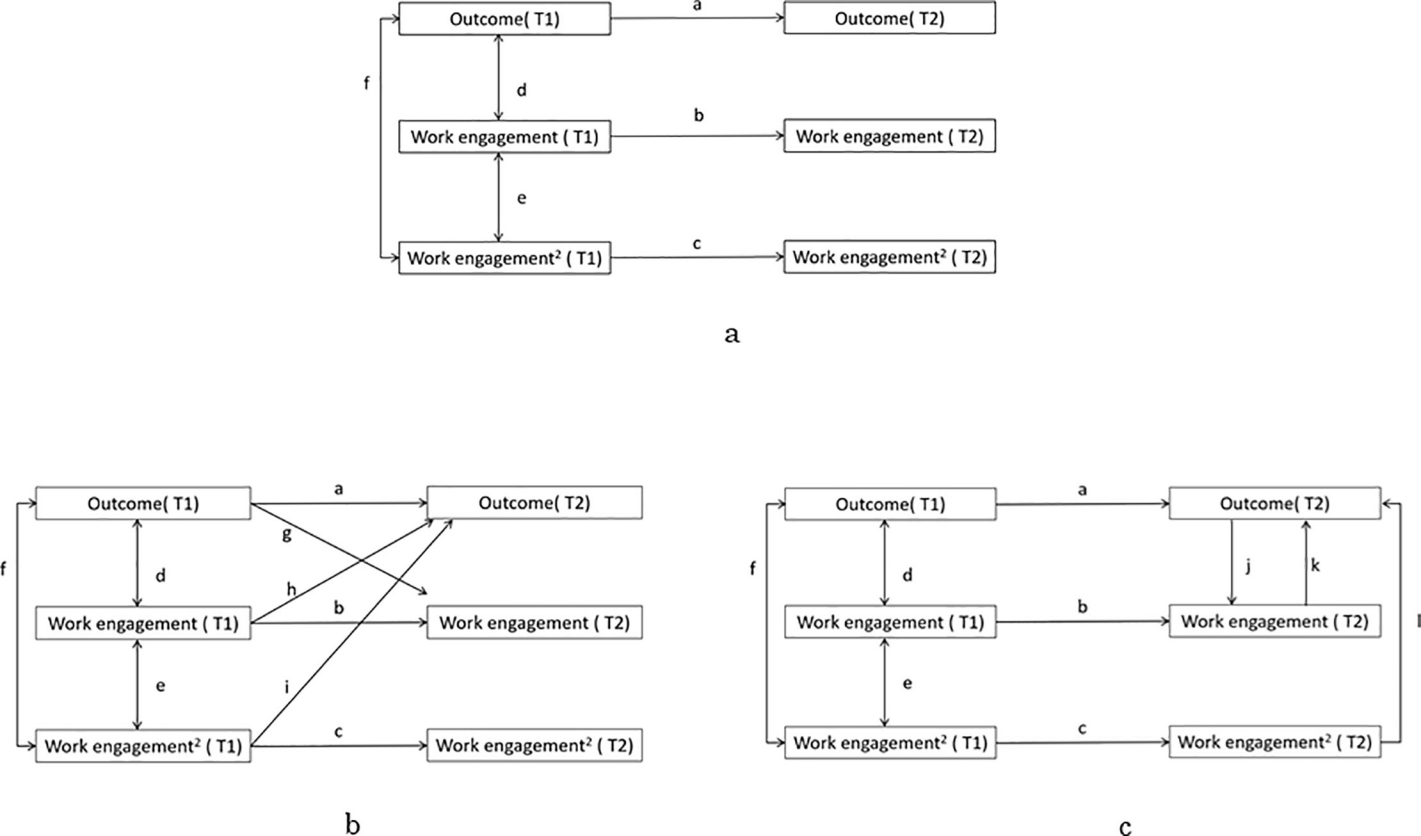
**Models for testing longitudinal relations between work engagement and outcomes in stability model (Fig 1A), lagged model (Fig 1B), and concurrent model (Fig 1C).** In Fig 1A, arrows a, b, c are the stability coefficients, and arrows d, e, f are the covariances at Time 1 (T1). In Fig 1B, arrow a, b, c are the stability coefficients, and arrows d, e, f are the covariance at Time 1 (T1); arrows g, h, and I are the cross-lagged coefficients. In Fig 1C, arrow a, b, c are the stability coefficients, and arrows d, e, f are the covariance at Time 1 (T1); arrows j, k, and l are concurrent coefficients at Time 2 (T2).

Fig 1 shows the model that includes six variables: a particular outcome at T1 and T2 and the linear and quadratic terms of work engagement. The quadratic terms were added to the model to test for the expected curvilinear relationship between work engagement and the particular outcome involved. The linear terms were centered before calculating the quadratic terms to correct for multicollinearity [49].

In the so-called stability model (Fig 1A), only the stability coefficients (arrows a, b, and c) and the correlations at T1 (arrows d, e, and f) are included. In each of the six tested models, the stability model was adjusted by releasing one or two additional paths. In lagged models (Fig 1B), these paths are outcome (T1) → work engagement (T2) (arrow g), work engagement (T1) → outcome (T2) (arrow h), and work engagement (T1) → outcome (T2) & work engagement^2^ (T1) → outcome (T2) (arrows h and i). In concurrent models (Fig 1C), these paths are described as outcome (T2) → work engagement (T2) (arrow j), work engagement (T2) → outcome (T2) (arrow k), and work engagement (T2) → outcome (T2) & work engagement^2^ (T2) → outcome (T2) (arrows k and l). Please note that, in the curvilinear models, two paths were released: the linear term and the curvilinear term, as recommended when testing for curvilinear effects with multiple regression [49]. For a curvilinear effect to be acknowledged, the model encompassing the curvilinear effect must significantly improve the fit of the model that only includes the linear term.

## Results

### Descriptive statistics

First, we conducted confirmatory factor analyses to examine the discriminant validity of the constructs of interest in each survey (T1 and T2). Results indicated that a four-factor model was superior (χ^2^[23] = 572.78 and 513.20, goodness of fit index [GFI] = .94 and .95, adjusted GFI [AGFI] = .88 and .89, comparative fit index [CFI] = .95 and .96, root mean square error of approximation [RMSEA] = .11 and 10, for T1 and T2, respectively) to the one-factor model (χ^2^[27) = 4441.08 and 4499.38, GFI = .62 and .62, AGFI = .36 and .37, CFI = .60 and .60, RMSEA = .29 and .29 for T1 and T2, respectively). This four-factor model was also statistically superior to a one-factor model, Δχ^2^(4) = 3868.30, *p* < .001 for T1 and Δχ^2^(4) = 3986.18, *p* < .001 for T2.

Given that creative performance and in-role performance are substantially correlated (r = .46 at T1 and r = .45 at T2) as well as work engagement and creative performance (r = .47 at T1 and r = .46 at T2: see Table 1), we additionally tested two three-factor models, whereby creative performance and in-role performance were treated as one latent factor (Model A) and work engagement and creative performance were treated as one latent factor (Model B), respectively. Regarding Model A, the four-factor model was superior to Model A (χ^2^ [26] = 1057.36 and 1079.37, GFI = .88 and .88, AGFI = .80 and .79, CFI = .91 and .91, RMSEA = .14 and .14 for T1 and T2, respectively). The fit of the four-factor model to the data was also statistically superior to Model A, Δχ^2^(3) = 484.58, p < .001 for T1 and Δχ2(3) = 566.17, p < .001 for T2. Regarding Model B, the four-factor model was superior to Model B (χ^2^ [25] = 819.50 and 729.68, GFI = .92 and .93, AGFI = .85 and .87, CFI = .93 and .94, RMSEA = .13 and .12 for T1 and T2, respectively). Also, the fit of the four-factor model to the data was statistically superior to Model B, Δχ^2^(2) = 246.72, p < .001 for T1 and Δχ^2^(2) = 216.48, p < .001 for T2. These results suggest that the four-factor model also performed better than the three-factor models. All subscales of the four–factor model loaded on their latent constructs with standardized loadings above .71 and .69 for T1 and T2, respectively.

**Table 1:** Means, standard deviations, and correlations between the variables (N = 1,967).

Variable		Range	Mean	SD	1	2	3	4	5	6	7
1	EN (T1)	0–6	2.81	1.22							
2	EN (T2)	0–6	2.80	1.22	.76						
3	PD (T1)	1–4	1.99	.68	-.32	-.26					
4	PD (T2)	1–4	1.97	.69	-.26	-.30	.66				
5	IP (T1)	1–4	3.13	.56	.34	.27	-.12	-.11			
6	IP (T2)	1–4	3.10	.56	.30	.37	-.10	-.12	.46		
7	CB (T1)	1–4	2.54	.75	.47	.40	-.12	-.08	.46	.31	
8	CB (T2)	1–4	2.55	.73	.39	.46	-.09	-.10	.26	.45	.61

Note: All correlation coefficients were significant at .001 level.

EN = Work engagement, PD = Psychological distress IP = In-role performance, CB = Creative behavior; SD = Standard Deviation; T1 = Time 1, T2 = Time 2.

Table 1 shows the means, standard deviations, and correlations between the study variables. Work engagement (T1) was associated negatively with psychological distress (T2) and positively with in-role performance (T2) and creative behavior (T2).

### Two-wave panel models

Table 2 shows the fit of the models examining the longitudinal relations between work engagement and psychological distress. As for the lagged models, M3 shows the best fit, which is superior to that of M1 (stability model); Δχ^2^(1) = 8.05, *p* < .001. This model (M3) showed a linear relation from work engagement at T1 to psychological distress at T2 longitudinally (*β* = −.05, *p* < .01). The relation did not change even after controlling for demographic variables such as age, gender and occupation (*β* = −.05, *p* < .01). Therefore, Hypothesis 1a was not confirmed.

**Table 2:** Longitudinal relations between work engagement and psychological distress (N = 1,967).

Model		AGFI	CFI	RMSEA	χ^2^	df	p	Model comparison	Δχ^2^	Δdf	p
[M1]	Stability model	.96	.98	.07	102.67	9	.00				
	Lagged models										
[M2]	PSY (T1) —> EN (T2)	.96	.98	.08	100.75	8	.00	M1 vs M2	1.92	1	.17
[M3]	EN (T1) —> PSY (T2)	.96	.98	.07	94.62	8	.00	M1 vs M3	8.05	1	.00
[M4]	EN (T1) —> PSY (T2) & EN2 (T1) —> PSY (T2)	.95	.98	.08	94.42	7	.00	M1 vs M4	8.25	2	.02
								M3 vs M4	0.20	1	.65
	Concurrent models										
[M5]	PSY (T2) —> EN (T2)	.98	.99	.05	53.22	8	.00	M1 vs M5	49.45	1	.00
[M6]	EN (T2) —> PSY (T2)	.98	.99	.05	47.05	8	.00	M1 vs M6	55.62	1	.00
[M7]	EN (T2) —> PSY (T2) & EN2 (T2) —> PSY (T2)	.98	.99	.05	39.60	7	.00	M1 vs M7	63.07	2	.00
								M6 vs M7	7.45	1	.01

Note. EN = Work engagement, PD = Psychological distress; T1 = Time 1, T2 = Time 2.

**Fig 2:**
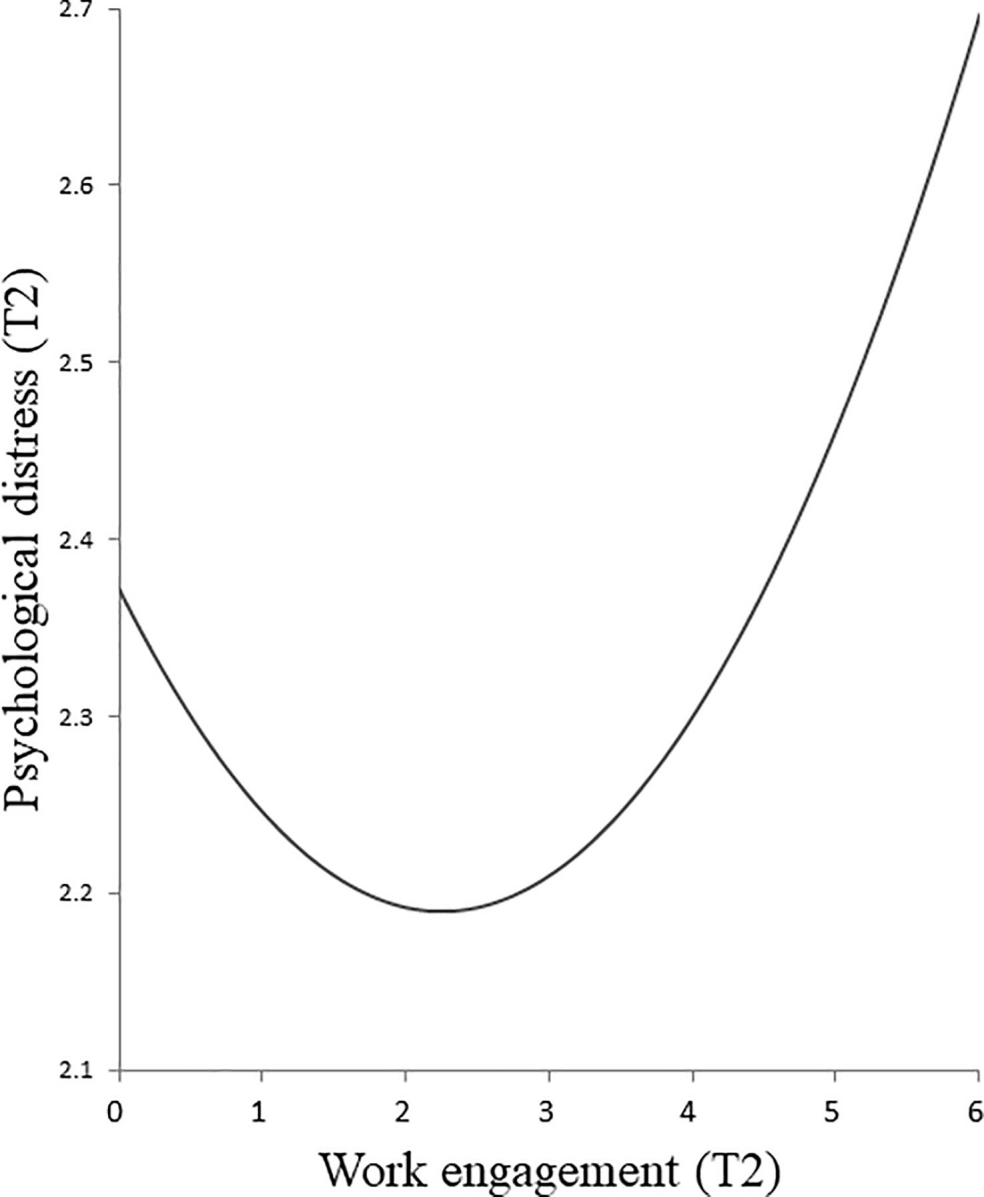
Curvilinear relation between work engagement at Time 2 (T2) and psychological distress at Time 2 (T2).

As for the concurrent models, M7 shows the best fit, which is superior to those of M1 and M6 (the model with linear term); Δχ^2^(2) = 63.07, *p* < .001 and Δχ^2^(1) = 7.45, *p* < .01, respectively. This model (M7) showed concurrent linear and curvilinear relations from work engagement at T2 to psychological distress at T2 (*β* = −.13, *p* < .001, and *β* = .05, *p* < .01, respectively). These relations did not almost change even after controlling for demographic variables (*β* = −.13, *p* < .001, and *β* = .04, *p* < .01, respectively). Regarding the curvilinear relation between work engagement and psychological distress at T2, Fig 2 shows that the lowest level of psychological distress was found at intermediate levels of work engagement. Put differently, there is initially a negative relation between work engagement and psychological distress. However, at intermediate levels of work engagement, this negative relation fades, and, instead, a positive relation is more prominent. This relationship suggests a U-shaped pattern. Therefore, Hypothesis 1b was confirmed.

**Fig 3:**
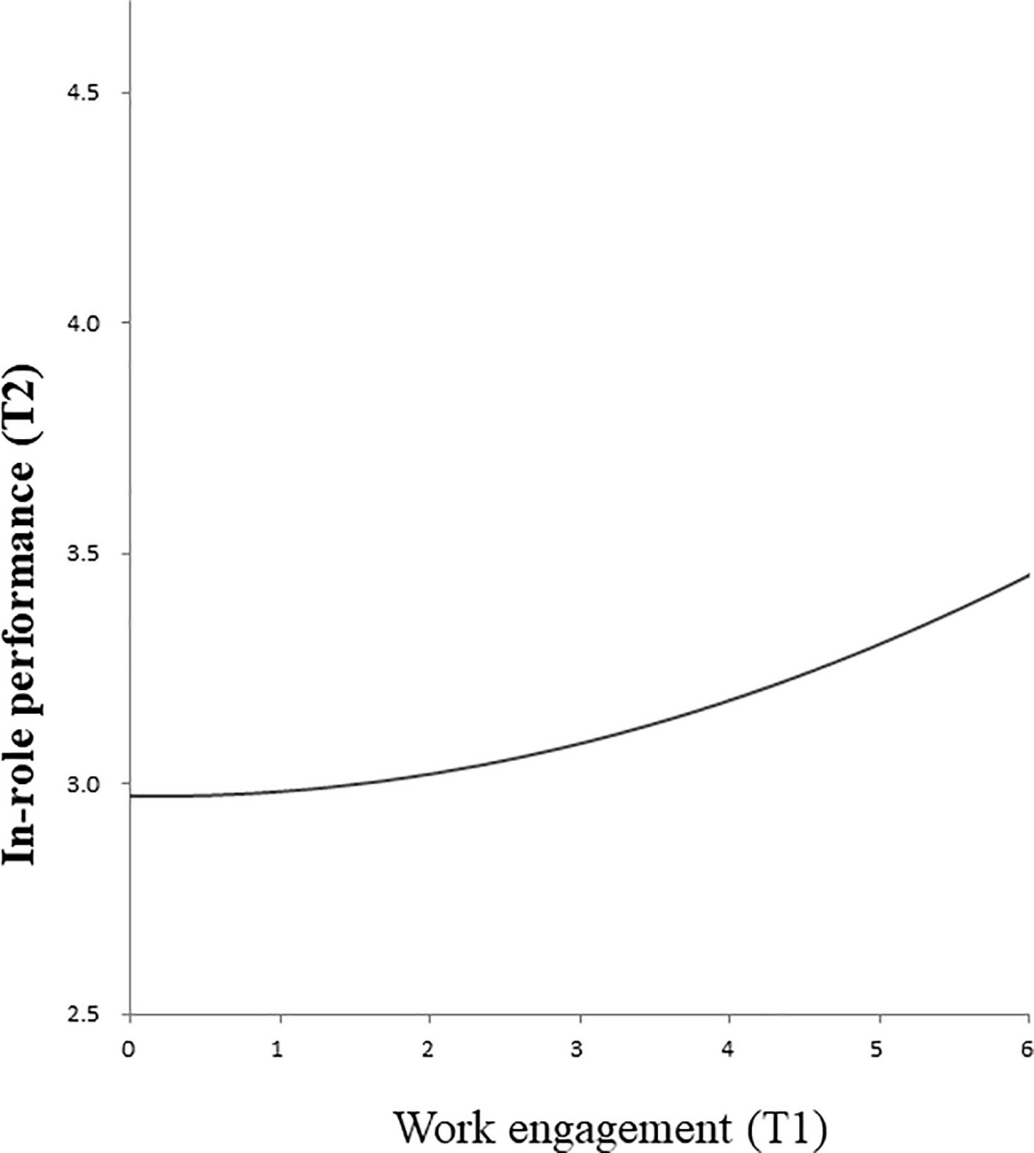
Curvilinear relation between work engagement at Time 1 (T1) and in-role performance at Time 2 (T2).

Table 3 shows the fit of the models examining the longitudinal relations between work engagement and in-role performance. As for the lagged models, the fit of M4 is superior to those of M1 (stability model) and M3 (the model with linear term); Δχ^2^(2) = 64.97, *p* < .001 and Δχ^2^(1) = 7.52, *p* < .05, respectively. This model (M4) showed lagged linear and curvilinear relations from work engagement at T1 to in-role performance at T2 (*β* = .16, *p* < .001, and *β* = .01, *p* < .01, respectively). These relations did not almost change even after controlling for demographic variables (*β* = .17, *p* < .001, and *β* = .06, *p* < .01, respectively). Fig 3 graphically displays this relation after controlling for in-role performance at T1. The figure shows that, the higher the levels of work engagement are at T1, the better the in-role performance is at T2. Although the results revealed a curvilinear relation between work engagement and in-role performance, contrary to our expectations this relation did not follow an inverted U-shape. Therefore, Hypothesis 2a was not confirmed.

**Table 3:** Longitudinal relations between work engagement and in-role performance (N = 1,967).

Model		AGFI	CFI	RMSEA	χ^2^	df	p	Model comparison	⊿χ^2^	⊿df	p
[M1]	Stability model	.92	.94	.11	212.70	9	.00				
	Lagged models										
[M2]	IP (T1) —> EN (T2)	.91	.94	.11	212.08	8	.00	M1 vs M2	0.62	1	.43
[M3]	EN (T1) —> IP (T2)	.94	.96	.10	155.25	8	.00	M1 vs M3	57.45	1	.00
[M4]	EN (T1) —> IP (T2) & EN2 (T1) —> IP (T2)	.93	.96	.10	147.73	7	.00	M1 vs M4	64.97	2	.00
								M3 vs M4	7.52	1	.01
	Concurrent models										
[M5]	IP (T2) —> EN (T2)	.95	.97	.08	106.72	8	.00	M1 vs M5	105.98	1	.00
[M6]	EN (T2) —> IP (T2)	.98	.99	.05	44.55	8	.00	M1 vs M6	168.16	1	.00
[M7]	EN (T2) —> IP (T2) & EN2 (T2) —> IP (T2)	.98	.99	.04	32.77	7	.00	M1 vs M7	179.93	2	.00
								M6 vs M7	11.78	1	.00

Note. EN = Work engagement, IP = In-role performance; T1 = Time 1, T2 = Time 2.

**Fig 4:**
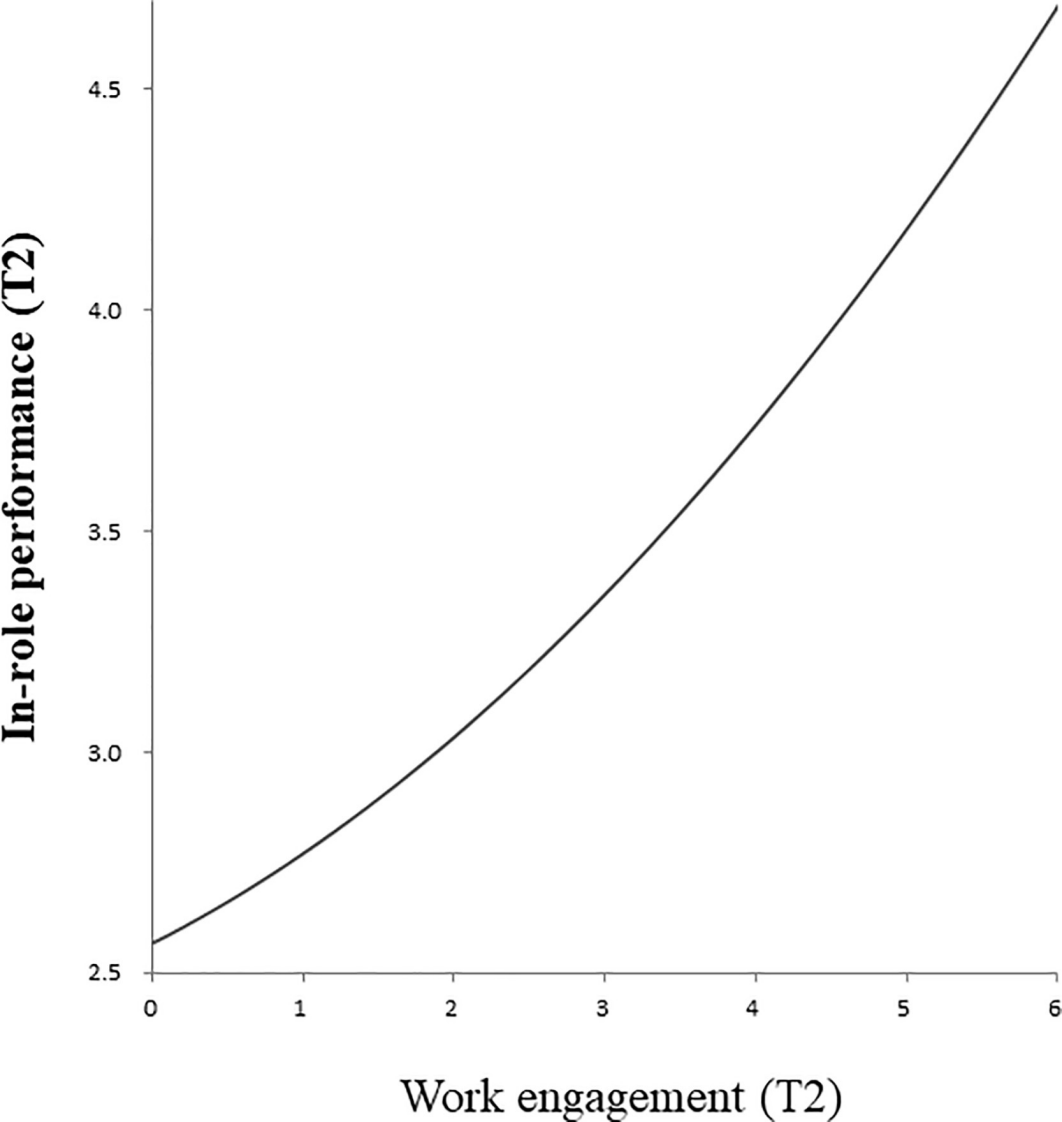
Curvilinear relation between work engagement at Time 2 (T2) and in-role performance at Time 2 (T2).

The fit of concurrent model M7 is superior to those of M1 (stability model) and M6 (the model with linear term); Δχ^2^(2) = 179.93, *p* < .001 and Δχ^2^(1) = 11.78, *p* < .01, respectively. This model (M7) showed concurrent linear and curvilinear relations between work engagement at T2 and in-role performance at T2 (*β* = .27, *p* < .001, and *β* = .07, *p* < .001, respectively). These relations did not almost change even after controlling for demographic variables (*β* = .28, *p* < .001, and *β* = .07, *p* < .01, respectively). Fig 4 graphically displays this relation, indicating again that, the higher the levels of work engagement are at T2, the better the in-role performance is at T2. Because this relation did not follow an inverted U-shape, Hypothesis 2b was not also confirmed.

Table 4 shows the fit of the models examining the longitudinal relations between work engagement and creative behavior. As for lagged models, M3 shows a superior fit compared to M1 (stability model); Δχ^2^(1) = 45.65, *p* < .001. This model (M3) showed a linear relation from work engagement at T1 to creative behavior at T2 longitudinally (*β* = .14, *p* < .001). This relation did not change even after controlling for demographic variables (*β* = .14, *p* < .001). Therefore, Hypothesis 3a was not confirmed.

**Table 4:** Longitudinal relations between work engagement and creative behavior (N = 1,967).

Model		AGFI	CFI	RMSEA	χ^2^	df	p	Model comparison	⊿χ^2^	⊿df	p
[M1]	Stability model	.91	.95	.12	244.00	9	.00				
	Lagged models										
[M2]	CB (T1) —> EN (T2)	.90	.95	.12	235.20	8	.00	M1 vs M2	8.80	1	.00
[M3]	EN (T1) —> CB (T2)	.92	.96	.11	198.35	8	.00	M1 vs M3	45.65	1	.00
[M4]	EN (T1) —> CB (T2) & EN2 (T1) —> CB (T2)	.91	.96	.12	197.94	7	.00	M1 vs M4	46.06	2	.00
								M3 vs M4	0.41	1	.52
	Concurrent models										
[M5]	CB (T2) —> EN (T2)	.96	.98	.07	83.48	8	.00	M1 vs M5	160.52	1	.00
[M6]	EN (T2) —> CB (T2)	.98	.99	.05	47.79	8	.00	M1 vs M6	196.21	1	.00
[M7]	EN (T2) —> CB (T2) & EN2 (T2) —> CB (T2)	.98	.99	.05	46.57	7	.00	M1 vs M7	197.43	2	.00
								M6 vs M7	1.21	1	.27

Note. EN = Work engagement, CB = Creative behavior; T1 = Time 1, T2 = Time 2.

As for concurrent models, M6 shows a superior fit compared to M1 (stability model); Δχ^2^(1) = 196.21, *p* < .001. This model (M6) showed a linear relation from work engagement at T2 to creative behavior at T2 concurrently (*β* = .27, *p* < .001). This relation did not almost change even after controlling for demographic variables (*β* = .28, *p* < .001). Therefore, Hypothesis 3b was not also confirmed.

To summarize (Table 5), work engagement had (1) longitudinal and linear, and (2) concurrent and curvilinear (U-shaped) relations with psychological distress. It also had (3) longitudinal and curvilinear, and (4) concurrent and curvilinear relations with in-role performance. Furthermore, it had (5) longitudinal and linear, and (6) concurrent and linear relations with creative behavior.

**Table 5:** Pattern of the relation between work engagement and outcomes.

Outcome	Pattern of the relation	
	Lagged model	Concurrent model
Psychological distress	(1) Longitudinal; Linear	(2) Concurrent; Curvilinear (U-shaped) (Fig 2)
In-role performance	(3) Longitudinal; Curvilinear (Fig 3)	(4) Concurrent; Curvilinear (Fig 4)
Creative behavior	(5) Longitudinal; Linear	(6) Concurrent; Linear

## Discussion

Most studies report a positive relationship between work engagement and health and job performance, but, occasionally, a “dark side” of engagement was also uncovered. Therefore, it seems that engagement is both positively and negatively associated with health and job performance, hence suggesting a curvilinear relationship. The current study took up the challenge to investigate this curvilinear relationship—whether work engagement has (1) a U-shaped curvilinear relation with psychological distress or (2) an inverted U-shaped curvilinear relation with job performance.

As for the relation between work engagement and psychological distress, we found not only a linear but also a curvilinear relationship in the concurrent model. The latter means that a negative relation was found for low levels of work engagement, but this negative relation faded at intermediate levels, and, at higher levels, a positive relation was observed (Fig 2). This result suggests that the favorable effect of work engagement on mental health may reach an upper limit, after which it levels off, and adverse effects may occur. This pattern resembles the AD effect of the Vitamin model [31, 32]. High levels of arousal go along with high levels of work engagement [51], which may counteract a further beneficial effect of work engagement on psychological distress as high levels of activation are known to be related to distress [52].

It is important to note that the lagged model showed only linear relations between work engagement and psychological distress; work engagement at T1 was negatively related to psychological distress seven months later (T2). This result contrasts with the concurrent model, which showed both linear *and* curvilinear relations at T2. Taken together, these results suggest that, although very high levels of work engagement may have a detrimental effect on mental health in a short run, this may disappear and even turn into a positive effect on mental health in the long run. Since high levels of work engagement go along with high levels of arousal [51], high levels may initially be counterproductive before reaping benefits. Engaged employees craft their jobs in such a way that they increase their own job resources [53]. This means that, in terms of the Conservation of Resources theory [54], they accumulate their resources and increase their resource pool. In turn, the accumulation of resources is related to increased (mental) health [54]. Therefore, high levels of work engagement may contribute to improvement of mental health in the long run. Future research must clarify how long it takes before high levels of work engagement may be beneficial to mental health.

As for the relation between work engagement and in-role performance, we found not only a linear but also a curvilinear relationship for the concurrent as well as the lagged models. A closer look at the curvilinear fit of models (Figs 3 and 4) reveals that, the higher the levels of work engagement are, the better the in-role performance is. This result suggests that even excessively high levels of work engagement are not detrimental; on the contrary, these are related positively to in-role performance. However, given that the curve in Fig 3 (lagged model) is flatter than the one in Fig 4 (concurrent model), the favorable effects on in-role performance seem to become weaker over time.

In terms of the relation with creative behavior, contrary to our expectations, we found a linear instead of a curvilinear relation across two models (i.e., concurrent and lagged models); higher levels of work engagement were associated with higher levels of creative behavior. Thus, it seems that there is neither a deliberating nor a leveling-off effect of work engagement on creative behavior. This agrees with recent insights dismissing the popular Yerks-Dodson Law that stipulates that increasing effort and motivation is beneficial to performance until some optimum level is reached, after which performance will decline [55]. However, it should be noted that the lagged effect of work engagement at T1 on creative behavior at T2 is weaker than the concurrent effect at T2. This suggests that the favorable effect of work engagement on creative behavior weakens over time.

### Limitations

Next to several strengths such as well-designed statistical analyses (i.e., two-wave panel model) to specify the directions and the time frame of the relations between work engagement and various outcomes, the current study also has some limitations.

First, dropout analyses showed that our sample is older and includes more men compared with those who dropped out between T1 and T2, suggesting that older and male employees had higher interest in participating in this multi-wave survey. Furthermore, our sample was also more highly educated compared to the Japanese population. Although our results can be applicable across age, gender, educational attainment, and occupation at least in our sample, generalization of the current results to other sample which include younger, more female, and less educated employees awaits further empirical examination.

Second, we used self-report survey data. Therefore, common method variance might have affected the results, suggesting that the true associations between variables might be weaker than those observed in our study. We conducted confirmatory factor analysis to examine the discriminant validity of the study variables. The results of confirmatory factor analysis showed that a four-factor model was superior to a one-factor model. This suggests that common method variance is not of great concern and, thus, is unlikely to confound the interpretations of the results. Please note that Siemsen et al. [56] showed that “common method variance deflates regression estimates of quadratic effects” [p16]. Hence, significant quadratic effects of work engagement in this study provide strong evidence of a curvilinear effect.

Third, our data were collected via the Internet, which requires caution regarding the generalizability of our findings. It has been claimed that the socioeconomic and educational status of the average Internet user is usually higher than that of the general population [57, 58]. Indeed, our participants were more highly educated than those completing nationwide paper-and-pencil surveys in Japan [42]. It is also claimed that, in Internet samples, younger participants are overrepresented [59]. Although we could not check differences between the initial (i.e., research volunteers registered in an Internet survey company) and final samples, self-selection might be a limitation of the present study.

Finally, although cross-lagged panel models specify the direction and time frame of the relations between work engagement and outcomes, the underlying mechanisms that govern these associations remain unknown. By using a three-wave dataset that allows study of mediation effects, the underlying psychological mechanisms may be uncovered.

### Directions for future research

Work engagement has been predominantly studied using the conceptual framework of the Job Demands–Resources (JD-R) model [60]. This model assumes that work engagement plays a crucial, mediating role in a motivational process sparked by job resources and leads—via work engagement—to positive outcomes such as health and job performance. The findings of the current study suggest that work engagement plays a different role in motivation enhancement than in health enhancement. More specifically, highly engaged employees may maintain high job performance whereas positive effects on mental health seem to diminish after a certain level of engagement (but only in the short run). Interestingly, although high levels of work engagement may have detrimental effects on mental health in the short run, its detrimental effect may disappear and even improve mental health in the long run. Future studies on the JD-R model should include curvilinear relationships between work engagement and individual outcomes, such as health and job performance. In addition, future research should investigate both lagged and concurrent effects to examine the time frame of the relations between the variables.

## Conclusions

The current study challenged the assumption of linearity and investigated curvilinear relationship between work engagement and psychological distress and job performance. Our results demonstrated that work engagement co-varies with psychological distress and job performance over time. However, different patterns were observed for health and job performance. Leveling-off and adverse effects of high work engagement were observed for psychological distress whereas no leveling-off effect was observed for job performance. Except for short-term effect on psychological distress, no dark side of work engagement was observed.
